# Supplementary material for: Stigma associated with cutaneous and mucocutaneous leishmaniasis: A systematic review
Source: PLoS Negl Trop Dis. 2023 Dec 28;17(12):e0011818. doi: 10.1371/journal.pntd.0011818 (PMC10781107; doi:10.1371/journal.pntd.0011818)
Supplement: S1 File — (PDF) [file pntd.0011818.s001.pdf]

Cochrane database search

Search Name:
Date Run: 10/03/2023 09:19:43
Comment:

ID Search Hits
#1 (("Stigma*) OR ("discriminat*") OR ("stereotyp*") OR ("negative attitude") OR ("psychosocial impact") OR ("psychosocial burden") OR ("social consequences") OR ("stigmatizing effect") OR ("scar*") OR ("disfigure*") OR ("self-stigma) OR ("label avoidance") OR ("disgrace") OR ("shame") OR ("perception") OR ("rejection")):ti,ab,kw (Word variations have been searched) 1103082
#2 (("Dermal leishman*") OR ("cutaneous leishman*") OR ("oriental sore") OR ("Uta") OR ("Chiclero ulcer") OR ("tropical sore") OR ("Bagdad boil") OR ("Baghdad boil") OR ("Bauer ulcer") OR ("Delhi boil") OR ("Aleppo boil") OR ("Aleppo button") OR ("Jericho boil") OR ("one year sore") OR ("one year ulcer") OR ("tegumentary leishman*") OR ("Biskra button") OR ("Biskra nodule") OR ("Calcutta ulcer") OR ("Jericho button") OR ("Kandahar sore") OR ("Lahore sore") OR ("Oriental button") OR ("Pian bois") OR ("Old World leishmaniasis") OR ("Mucosal Leishman*") OR ("Mucocutaneous Leishman*") OR ("muco cutaneous Leishman*") OR ("espundia") OR ("nasopharyngeal Leishman*") OR ("New World leishmaniasis")):ti,ab,kw (Word variations have been searched) 47
#3 #1 AND #2 32

#	Query	Limiters/Expanders	Last Run Via	Results
S13	S5 AND S12	Expanders - Apply equivalent subjects<br>Search modes - Boolean/Phrase	Interface - EBSCOhost Research Databases<br>Search Screen - Advanced Search<br>Database - Abstracts in Social Gerontology;Academic Search Complete;America: History & Life;APA PsycArticles;APA PsycInfo;Art & Architecture Source;British Education Index;Business Source Ultimate;Child Development & Adolescent Studies;CINAHL Plus with Full Text;Criminal Justice Abstracts;eBook Collection (EBSCOhost);EconLit with Full Text;Education Abstracts (H.W. Wilson);Educational Administration Abstracts;ERIC;European Views of the Americas: 1493 to 1750;Film & Television Literature Index with Full Text;GreenFILE;Historical Abstracts;Index to Legal Periodicals and Books (H.W. Wilson);International Bibliography of Theatre & Dance with Full Text;Library, Information Science & Technology Abstracts;MathSciNet via EBSCOhost;MLA Directory of Periodicals;MLA International Bibliography;OpenDissertations;Regional Business News;SocINDEX with Full Text;SPORTDiscus with Full Text;Wildlife & Ecology Studies Worldwide	606
S12	S6 OR S7 OR S8 OR S9 OR S10 OR S11	Expanders - Apply equivalent subjects<br>Search modes - Boolean/Phrase	Interface - EBSCOhost Research Databases<br>Search Screen - Advanced Search<br>Database - Abstracts in Social Gerontology;Academic Search Complete;America: History & Life;APA PsycArticles;APA PsycInfo;Art & Architecture Source;British Education Index;Business Source Ultimate;Child Development & Adolescent Studies;CINAHL Plus with Full Text;Criminal Justice Abstracts;eBook Collection (EBSCOhost);EconLit with Full Text;Education Abstracts (H.W. Wilson);Educational Administration Abstracts;ERIC;European Views of the Americas: 1493 to 1750;Film & Television Literature Index with Full Text;GreenFILE;Historical Abstracts;Index to Legal Periodicals and Books (H.W. Wilson);International Bibliography of Theatre & Dance with Full Text;Library, Information Science & Technology Abstracts;MathSciNet via EBSCOhost;MLA Directory of Periodicals;MLA International Bibliography;OpenDissertations;Regional Business News;SocINDEX with Full Text;SPORTDiscus with Full Text;Wildlife & Ecology Studies Worldwide	12,583
S11	AB Old World leishmaniasis OR AB Mucosal Leishman* OR AB Mucocutaneous Leishman* OR AB muco cutaneous Leishman* OR AB espundia OR AB nasopharyngeal Leishman* OR AB New World leishmaniasis OR AB American leishmaniasis OR AB leishmaniasis americana	Expanders - Apply equivalent subjects<br>Search modes - Boolean/Phrase	Interface - EBSCOhost Research Databases<br>Search Screen - Advanced Search<br>Database - Abstracts in Social Gerontology;Academic Search Complete;America: History & Life;APA PsycArticles;APA PsycInfo;Art & Architecture Source;British Education Index;Business Source Ultimate;Child Development & Adolescent Studies;CINAHL Plus with Full Text;Criminal Justice Abstracts;eBook Collection (EBSCOhost);EconLit with Full Text;Education Abstracts (H.W. Wilson);Educational Administration Abstracts;ERIC;European Views of the Americas: 1493 to 1750;Film & Television Literature Index with Full Text;GreenFILE;Historical Abstracts;Index to Legal Periodicals and Books (H.W. Wilson);International Bibliography of Theatre & Dance with Full Text;Library, Information Science & Technology Abstracts;MathSciNet via EBSCOhost;MLA Directory of Periodicals;MLA International Bibliography;OpenDissertations;Regional Business News;SocINDEX with Full Text;SPORTDiscus with Full Text;Wildlife & Ecology Studies Worldwide	1,367
S10	TI Old World leishmaniasis OR TI Mucosal Leishman* OR TI Mucocutaneous Leishman* OR TI muco cutaneous Leishman* OR TI espundia OR TI nasopharyngeal Leishman* OR TI New World leishmaniasis OR TI American leishmaniasis OR TI leishmaniasis americana	Expanders - Apply equivalent subjects<br>Search modes - Boolean/Phrase	Interface - EBSCOhost Research Databases<br>Search Screen - Advanced Search<br>Database - Abstracts in Social Gerontology;Academic Search Complete;America: History & Life;APA PsycArticles;APA PsycInfo;Art & Architecture Source;British Education Index;Business Source Ultimate;Child Development & Adolescent Studies;CINAHL Plus with Full Text;Criminal Justice Abstracts;eBook Collection (EBSCOhost);EconLit with Full Text;Education Abstracts (H.W. Wilson);Educational Administration Abstracts;ERIC;European Views of the Americas: 1493 to 1750;Film & Television Literature Index with Full Text;GreenFILE;Historical Abstracts;Index to Legal Periodicals and Books (H.W. Wilson);International Bibliography of Theatre & Dance with Full Text;Library, Information Science & Technology Abstracts;MathSciNet via EBSCOhost;MLA Directory of Periodicals;MLA International Bibliography;OpenDissertations;Regional Business News;SocINDEX with Full Text;SPORTDiscus with Full Text;Wildlife & Ecology Studies Worldwide	699
S9	TI Jericho boil OR TI one year sore OR TI one year ulcer OR TI tegumentary leishman* OR TI Biskra button OR TI Biskra nodule OR TI Calcutta ulcer OR TI Jericho button OR TI Kandahar sore OR TI Lahore sore OR TI Oriental button OR TI Pian bois	Expanders - Apply equivalent subjects<br>Search modes - Boolean/Phrase	Interface - EBSCOhost Research Databases<br>Search Screen - Advanced Search<br>Database - Abstracts in Social Gerontology;Academic Search Complete;America: History & Life;APA PsycArticles;APA PsycInfo;Art & Architecture Source;British Education Index;Business Source Ultimate;Child Development & Adolescent Studies;CINAHL Plus with Full Text;Criminal Justice Abstracts;eBook Collection (EBSCOhost);EconLit with Full Text;Education Abstracts (H.W. Wilson);Educational Administration Abstracts;ERIC;European Views of the Americas: 1493 to 1750;Film & Television Literature Index with Full Text;GreenFILE;Historical Abstracts;Index to Legal Periodicals and Books (H.W. Wilson);International Bibliography of Theatre & Dance with Full Text;Library, Information Science & Technology Abstracts;MathSciNet via EBSCOhost;MLA Directory of Periodicals;MLA International Bibliography;OpenDissertations;Regional Business News;SocINDEX with Full Text;SPORTDiscus with Full Text;Wildlife & Ecology Studies Worldwide	227
S8	AB Jericho boil OR AB one year sore OR AB one year ulcer OR AB tegumentary leishman* OR AB Biskra button OR AB Biskra nodule OR AB Calcutta ulcer OR AB Jericho button OR AB Kandahar sore OR AB Lahore sore OR AB Oriental button OR AB Pian bois	Expanders - Apply equivalent subjects<br>Search modes - Boolean/Phrase	Interface - EBSCOhost Research Databases<br>Search Screen - Advanced Search<br>Database - Abstracts in Social Gerontology;Academic Search Complete;America: History & Life;APA PsycArticles;APA PsycInfo;Art & Architecture Source;British Education Index;Business Source Ultimate;Child Development & Adolescent Studies;CINAHL Plus with Full Text;Criminal Justice Abstracts;eBook Collection (EBSCOhost);EconLit with Full Text;Education Abstracts (H.W. Wilson);Educational Administration Abstracts;ERIC;European Views of the Americas: 1493 to 1750;Film & Television Literature Index with Full Text;GreenFILE;Historical Abstracts;Index to Legal Periodicals and Books (H.W. Wilson);International Bibliography of Theatre & Dance with Full Text;Library, Information Science & Technology Abstracts;MathSciNet via EBSCOhost;MLA Directory of Periodicals;MLA International Bibliography;OpenDissertations;Regional Business News;SocINDEX with Full Text;SPORTDiscus with Full Text;Wildlife & Ecology Studies Worldwide	705
S7	AB Dermal leishman* OR AB cutaneous leishman* OR AB oriental sore OR AB Uta OR AB Chiclero ulcer OR AB tropical sore OR AB Bagdad boil OR AB Baghdad boil OR AB Bauer ulcer OR AB Delhi boil OR AB Aleppo boil OR AB Aleppo button	Expanders - Apply equivalent subjects<br>Search modes - Boolean/Phrase	Interface - EBSCOhost Research Databases<br>Search Screen - Advanced Search<br>Database - Abstracts in Social Gerontology;Academic Search Complete;America: History & Life;APA PsycArticles;APA PsycInfo;Art & Architecture Source;British Education Index;Business Source Ultimate;Child Development & Adolescent Studies;CINAHL Plus with Full Text;Criminal Justice Abstracts;eBook Collection (EBSCOhost);EconLit with Full Text;Education Abstracts (H.W. Wilson);Educational Administration Abstracts;ERIC;European Views of the Americas: 1493 to 1750;Film & Television Literature Index with Full Text;GreenFILE;Historical Abstracts;Index to Legal Periodicals and Books (H.W. Wilson);International Bibliography of Theatre & Dance with Full Text;Library, Information Science & Technology Abstracts;MathSciNet via EBSCOhost;MLA Directory of Periodicals;MLA International Bibliography;OpenDissertations;Regional Business News;SocINDEX with Full Text;SPORTDiscus with Full Text;Wildlife & Ecology Studies Worldwide	9,852
S6	TI Dermal leishman* OR TI cutaneous leishman* OR TI oriental sore OR TI Uta OR TI Chiclero ulcer OR TI tropical sore OR TI Bagdad boil OR TI Baghdad boil OR TI Bauer ulcer OR TI Delhi boil OR TI Aleppo boil OR TI Aleppo button	Expanders - Apply equivalent subjects<br>Search modes - Boolean/Phrase	Interface - EBSCOhost Research Databases<br>Search Screen - Advanced Search<br>Database - Abstracts in Social Gerontology;Academic Search Complete;America: History & Life;APA PsycArticles;APA PsycInfo;Art & Architecture Source;British Education Index;Business Source Ultimate;Child Development & Adolescent Studies;CINAHL Plus with Full Text;Criminal Justice Abstracts;eBook Collection (EBSCOhost);EconLit with Full Text;Education Abstracts (H.W. Wilson);Educational Administration Abstracts;ERIC;European Views of the Americas: 1493 to 1750;Film & Television Literature Index with Full Text;GreenFILE;Historical Abstracts;Index to Legal Periodicals and Books (H.W. Wilson);International Bibliography of Theatre & Dance with Full Text;Library, Information Science & Technology Abstracts;MathSciNet via EBSCOhost;MLA Directory of Periodicals;MLA International Bibliography;OpenDissertations;Regional Business News;SocINDEX with Full Text;SPORTDiscus with Full Text;Wildlife & Ecology Studies Worldwide	5,073
S5	S1 OR S2 OR S3 OR S4	Expanders - Apply equivalent subjects<br>Search modes - Boolean/Phrase	Interface - EBSCOhost Research Databases<br>Search Screen - Advanced Search<br>Database - Abstracts in Social Gerontology;Academic Search Complete;America: History & Life;APA PsycArticles;APA PsycInfo;Art & Architecture Source;British Education Index;Business Source Ultimate;Child Development & Adolescent Studies;CINAHL Plus with Full Text;Criminal Justice Abstracts;eBook Collection (EBSCOhost);EconLit with Full Text;Education Abstracts (H.W. Wilson);Educational Administration Abstracts;ERIC;European Views of the Americas: 1493 to 1750;Film & Television Literature Index with Full Text;GreenFILE;Historical Abstracts;Index to Legal Periodicals and Books (H.W. Wilson);International Bibliography of Theatre & Dance with Full Text;Library, Information Science & Technology Abstracts;MathSciNet via EBSCOhost;MLA Directory of Periodicals;MLA International Bibliography;OpenDissertations;Regional Business News;SocINDEX with Full Text;SPORTDiscus with Full Text;Wildlife & Ecology Studies Worldwide	3,317,020
S4	AB disgrace OR AB shame OR AB perception OR AB rejection	Expanders - Apply equivalent subjects<br>Search modes - Boolean/Phrase	Interface - EBSCOhost Research Databases<br>Search Screen - Advanced Search<br>Database - Abstracts in Social Gerontology;Academic Search Complete;America: History & Life;APA PsycArticles;APA PsycInfo;Art & Architecture Source;British Education Index;Business Source Ultimate;Child Development & Adolescent Studies;CINAHL Plus with Full Text;Criminal Justice Abstracts;eBook Collection (EBSCOhost);EconLit with Full Text;Education Abstracts (H.W. Wilson);Educational Administration Abstracts;ERIC;European Views of the Americas: 1493 to 1750;Film & Television Literature Index with Full Text;GreenFILE;Historical Abstracts;Index to Legal Periodicals and Books (H.W. Wilson);International Bibliography of Theatre & Dance with Full Text;Library, Information Science & Technology Abstracts;MathSciNet via EBSCOhost;MLA Directory of Periodicals;MLA International Bibliography;OpenDissertations;Regional Business News;SocINDEX with Full Text;SPORTDiscus with Full Text;Wildlife & Ecology Studies Worldwide	1,547,498
S3	AB Stigma* OR AB discriminat* OR AB stereotyp* OR AB negative attitude OR AB psychosocial impact OR AB psychosocial burden OR AB social consequences OR AB stigmatizing effect OR AB scar* OR AB disfigure* OR AB self-stigma OR AB label avoidance	Expanders - Apply equivalent subjects<br>Search modes - Boolean/Phrase	Interface - EBSCOhost Research Databases<br>Search Screen - Advanced Search<br>Database - Abstracts in Social Gerontology;Academic Search Complete;America: History & Life;APA PsycArticles;APA PsycInfo;Art & Architecture Source;British Education Index;Business Source Ultimate;Child Development & Adolescent Studies;CINAHL Plus with Full Text;Criminal Justice Abstracts;eBook Collection (EBSCOhost);EconLit with Full Text;Education Abstracts (H.W. Wilson);Educational Administration Abstracts;ERIC;European Views of the Americas: 1493 to 1750;Film & Television Literature Index with Full Text;GreenFILE;Historical Abstracts;Index to Legal Periodicals and Books (H.W. Wilson);International Bibliography of Theatre & Dance with Full Text;Library, Information Science & Technology Abstracts;MathSciNet via EBSCOhost;MLA Directory of Periodicals;MLA International Bibliography;OpenDissertations;Regional Business News;SocINDEX with Full Text;SPORTDiscus with Full Text;Wildlife & Ecology Studies Worldwide	1,526,238
S2	TI disgrace OR TI shame OR TI perception OR TI rejection	Expanders - Apply equivalent subjects<br>Search modes - Boolean/Phrase	Interface - EBSCOhost Research Databases<br>Search Screen - Advanced Search<br>Database - Abstracts in Social Gerontology;Academic Search Complete;America: History & Life;APA PsycArticles;APA PsycInfo;Art & Architecture Source;British Education Index;Business Source Ultimate;Child Development & Adolescent Studies;CINAHL Plus with Full Text;Criminal Justice Abstracts;eBook Collection (EBSCOhost);EconLit with Full Text;Education Abstracts (H.W. Wilson);Educational Administration Abstracts;ERIC;European Views of the Americas: 1493 to 1750;Film & Television Literature Index with Full Text;GreenFILE;Historical Abstracts;Index to Legal Periodicals and Books (H.W. Wilson);International Bibliography of Theatre & Dance with Full Text;Library, Information Science & Technology Abstracts;MathSciNet via EBSCOhost;MLA Directory of Periodicals;MLA International Bibliography;OpenDissertations;Regional Business News;SocINDEX with Full Text;SPORTDiscus with Full Text;Wildlife & Ecology Studies Worldwide	518,400
S1	TI Stigma* OR TI discriminat* OR TI stereotyp* OR TI negative attitude OR TI psychosocial impact OR TI psychosocial burden OR TI social consequences OR TI stigmatizing effect OR TI scar* OR TI disfigure* OR TI self-stigma OR TI label avoidance	Expanders - Apply equivalent subjects<br>Search modes - Boolean/Phrase	Interface - EBSCOhost Research Databases<br>Search Screen - Advanced Search<br>Database - Abstracts in Social Gerontology;Academic Search Complete;America: History & Life;APA PsycArticles;APA PsycInfo;Art & Architecture Source;British Education Index;Business Source Ultimate;Child Development & Adolescent Studies;CINAHL Plus with Full Text;Criminal Justice Abstracts;eBook Collection (EBSCOhost);EconLit with Full Text;Education Abstracts (H.W. Wilson);Educational Administration Abstracts;ERIC;European Views of the Americas: 1493 to 1750;Film & Television Literature Index with Full Text;GreenFILE;Historical Abstracts;Index to Legal Periodicals and Books (H.W. Wilson);International Bibliography of Theatre & Dance with Full Text;Library, Information Science & Technology Abstracts;MathSciNet via EBSCOhost;MLA Directory of Periodicals;MLA International Bibliography;OpenDissertations;Regional Business News;SocINDEX with Full Text;SPORTDiscus with Full Text;Wildlife & Ecology Studies Worldwide	353,868

Database:
Embase <1974 to 2023 March 09>

#	Query	Results from 11 Mar 2023
1	"Stigma*".ab,ti.	63,723
2	"discriminat*".ab,ti.	356,604
3	"stereotyp*".ab,ti.	35,338
4	negative attitude.ab,ti.	2,926
5	psychosocial impact.ab,ti.	3,892
6	psychosocial burden.ab,ti.	1,122
7	social consequences.ab,ti.	4,250
8	stigmatizing effect.ab,ti.	33
9	"scar*".ab,ti.	284,201
10	"disfigure*".ab,ti.	4,401
11	self-stigma.ab,ti.	1,430
12	label avoidance.ab,ti.	16
13	disgrace.ab,ti.	208
14	shame.ab,ti.	8,310
15	perception.ab,ti.	253,542
16	rejection.ab,ti.	144,344
17	1 or 2 or 3 or 4 or 5 or 6 or 7 or 8 or 9 or 10 or 11 or 12 or 13 or 14 or 15 or 16	1,120,120
18	"Dermal leishman*".ab,ti.	679
19	"cutaneous leishman*".ab,ti.	9,752
20	oriental sore.ab,ti.	73
21	Uta.ab,ti.	871
22	Chiclero ulcer.ab,ti.	4
23	tropical sore.ab,ti.	3
24	Bagdad boil.ab,ti.	1
25	Baghdad boil.ab,ti.	11
26	Bauer ulcer.ab,ti.	0
27	Delhi boil.ab,ti.	1
28	Aleppo boil.ab,ti.	12
29	Aleppo button.ab,ti.	3
30	Jericho boil.ab,ti.	0
31	one year sore.ab,ti.	3
32	one year ulcer.ab,ti.	6
33	"tegumentary leishman*".ab,ti.	718
34	Biskra button.ab,ti.	1
35	Biskra nodule.ab,ti.	0
36	Calcutta ulcer.ab,ti.	0
37	Jericho button.ab,ti.	0

38	Kandahar sore.ab,ti.	0
39	Lahore sore.ab,ti.	0
40	Oriental button.ab,ti.	1
41	Pian bois.ab,ti.	4
42	Old World leishmaniasis.ab,ti.	51
43	"Mucosal Leishman*".ab,ti.	435
44	"Mucocutaneous Leishman*".ab,ti.	669
45	"muco cutaneous Leishman*".ab,ti.	44
46	espundia.ab,ti.	31
47	"nasopharyngeal Leishman*".ab,ti.	0
48	New World leishmaniasis.ab,ti.	57
49	American leishmaniasis.ab,ti.	62
50	leishmaniasis americana.ab,ti.	8
51	18 or 19 or 20 or 21 or 22 or 23 or 24 or 25 or 26 or 27 or 28 or 29 or 30 or 31 or 32 or 33 or 34 or 35 or 36 or 37 or 38 or 39 or 40 or 41 or 42 or 43 or 44 or 45 or 46 or 47 or 48 or 49 or 50	12,460
52	17 and 51	757
53	limit 52 to (english or portuguese or spanish)	705

"Stigma*".ab,ti.
"discriminat*".ab,ti.
"stereotyp*".ab,ti.
negative attitude.ab,ti.
psychosocial impact.ab,ti.
psychosocial burden.ab,ti.
social consequences.ab,ti.
stigmatizing effect.ab,ti.
"scar*".ab,ti.
"disfigure*".ab,ti.
self-stigma.ab,ti.
label avoidance.ab,ti.
disgrace.ab,ti.
shame.ab,ti.
perception.ab,ti.
rejection.ab,ti.
1 or 2 or 3 or 4 or 5 or 6 or 7 or 8 or 9 or 10 or 11 or 12 or 13 or 14 or 15 or 16
"Dermal leishman*".ab,ti.
"cutaneous leishman*".ab,ti.
oriental sore.ab,ti.
Uta.ab,ti.
Chiclero ulcer.ab,ti.
tropical sore.ab,ti.
Bagdad boil.ab,ti.
Baghdad boil.ab,ti.
Bauer ulcer.ab,ti.
Delhi boil.ab,ti.
Aleppo boil.ab,ti.
Aleppo button.ab,ti.
Jericho boil.ab,ti.
one year sore.ab,ti.
one year ulcer.ab,ti.
"tegumentary leishman*".ab,ti.
Biskra button.ab,ti.
Biskra nodule.ab,ti.
Calcutta ulcer.ab,ti.

Jericho button.ab,ti.
Kandahar sore.ab,ti.
Lahore sore.ab,ti.
Oriental button.ab,ti.
Pian bois.ab,ti.
Old World leishmaniasis.ab,ti.
"Mucosal Leishman*".ab,ti.
"Mucocutaneous Leishman*".ab,ti.
"muco cutaneous Leishman*".ab,ti.
espundia.ab,ti.
"nasopharyngeal Leishman*".ab,ti.
New World leishmaniasis.ab,ti.
American leishmaniasis.ab,ti.
leishmaniasis americana.ab,ti.
18 or 19 or 20 or 21 or 22 or 23 or 24 or 25 or 26 or 27 or 28 or 29 or 30 or 31 or 32 or 33 or 34 or 35 or 36 or 37 or 38 or 39 or 40 or 41 or 42 or 43 or 44 or 45 or 46 or 47 or 48 or 49 or 50
17 and 51
limit 52 to (english or portuguese or spanish)

https://ovidsp.ovid.com/athens/ovidweb.cgi?T=JS&NEWS=N&PAGE=main&SHAREDSEARCHID=9STqL6eoGpYzir9DbQ0TpmYSGOvFjVt3pDfVXIM7HHk77JMmYXMkHpSdpQRjr7uw

Embase <1974 to 2023 March 09>

1	"Stigma*".ab,ti.	63723
2	"discriminat*".ab,ti.	356604
3	"stereotyp*".ab,ti.	35338
4	negative attitude.ab,ti.	2926
5	psychosocial impact.ab,ti.	3892
6	psychosocial burden.ab,ti.	1122
7	social consequences.ab,ti.	4250
8	stigmatizing effect.ab,ti.	33
9	"scar*".ab,ti.	284201
10	"disfigure*".ab,ti.	4401
11	self-stigma.ab,ti.	1430
12	label avoidance.ab,ti.	16
13	disgrace.ab,ti.	208
14	shame.ab,ti.	8310
15	perception.ab,ti.	253542
16	rejection.ab,ti.	144344
17	1 or 2 or 3 or 4 or 5 or 6 or 7 or 8 or 9 or 10 or 11 or 12 or 13 or 14 or 15 or 16	1120120
18	"Dermal leishman*".ab,ti.	679
19	"cutaneous leishman*".ab,ti.	9752
20	oriental sore.ab,ti.	73
21	Uta.ab,ti.	871
22	Chiclero ulcer.ab,ti.	4
23	tropical sore.ab,ti.	3
24	Bagdad boil.ab,ti.	1
25	Baghdad boil.ab,ti.	11
26	Bauer ulcer.ab,ti.	0
27	Delhi boil.ab,ti.	1

28 Aleppo boil.ab,ti. 12
29 Aleppo button.ab,ti. 3
30 Jericho boil.ab,ti. 0
31 one year sore.ab,ti. 3
32 one year ulcer.ab,ti. 6
33 "tegumentary leishman*".ab,ti. 718
34 Biskra button.ab,ti. 1
35 Biskra nodule.ab,ti. 0
36 Calcutta ulcer.ab,ti. 0
37 Jericho button.ab,ti. 0
38 Kandahar sore.ab,ti. 0
39 Lahore sore.ab,ti. 0
40 Oriental button.ab,ti. 1
41 Pian bois.ab,ti.4
42 Old World leishmaniasis.ab,ti. 51
43 "Mucosal Leishman*".ab,ti. 435
44 "Mucocutaneous Leishman*".ab,ti. 669
45 "muco cutaneous Leishman*".ab,ti. 44
46 espundia.ab,ti.31
47 "nasopharyngeal Leishman*".ab,ti. 0
48 New World leishmaniasis.ab,ti. 57
49 American leishmaniasis.ab,ti. 62
50 leishmaniasis americana.ab,ti. 8
51 18 or 19 or 20 or 21 or 22 or 23 or 24 or 25 or 26 or 27 or 28 or 29 or 30 or 31 or 32 or 33 or 34 or 35 or 36 or 37 or 38 or 39 or 40 or 41 or 42 or 43 or 44 or 45 or 46 or 47 or 48 or 49 or 50 12460
52 17 and 51 757
53 limit 52 to (english or portuguese or spanish) 705

Global Index Medicus database search

08/03/2023

tw:((tw:(tw:(dermal leishma*)) OR (tw:(cutaneous leishma*)) OR (tw:(oriental sore)) OR (tw:(uta)) OR (tw:(chiclero ulcer)) OR (tw:(tropical sore)) OR (tw:(baghdad boil)) OR (tw:(bauer ulcer)) OR (tw:(delhi boil)) OR (tw:(aleppo boil)) OR (tw:(aleppo button)) OR (tw:(jericho boil)) OR (tw:(balkh sore)) OR (tw:(one year sore)) OR (tw:(one year ulcer)) OR (tw:(tegumentary leishman*)) OR (tw:(biskra button)) OR (tw:(biskra nodule)) OR (tw:(calcutta ulcer)) OR (tw:(jericho button)) OR (tw:(kandahar sore)) OR (tw:(lahore sore)) OR (tw:(oriental button)) OR (tw:(pian bois)) OR (tw:(old world leishmaniasis)) OR (tw:(mucosal leishman*)) OR (tw:(mucocutaneous leishman*)) OR (tw:(muco cutaneous leishman*)) OR (tw:(espundia)) OR (tw:(nasopharyngeal leishman*)) OR (tw:(new world leishman*)) OR (tw:(an leishmaniasis)) OR (tw:(leishmaniasis americana))) AND (tw:(tw:(stigma*)) OR (tw:(discriminat*)) OR (tw:(stereotyp*)) OR (tw:(negative attitude)) OR (tw:(psychological)) OR (tw:(psychosocial)) OR (tw:(social consequences)) OR (tw:(scar*)) OR (tw:(disfigur*)) OR (tw:(self-stigma)) OR (tw:(label avoidance)) OR (tw:(disgrace)) OR (tw:(shame)) OR (tw:(perception)) OR (tw:(rejection)))) AND (la:("en" OR "pt" OR "es"))	122

Database:
Ovid MEDLINE(R) <1946 to February Week 5 2023>

#	Query	Results from 11 Mar 2023
1	"Stigma*".ab,ti.	40,541
2	"discriminat*".ab,ti.	242,711
3	"stereotyp*".ab,ti.	25,345
4	negative attitude.ab,ti.	1,709
5	psychosocial impact.ab,ti.	2,247
6	psychosocial burden.ab,ti.	559
7	social consequences.ab,ti.	2,855
8	stigmatizing effect.ab,ti.	27
9	"scar*".ab,ti.	171,517
10	"disfigure*".ab,ti.	2,661
11	self-stigma.ab,ti.	932
12	label avoidance.ab,ti.	15
13	disgrace.ab,ti.	152
14	shame.ab,ti.	5,278
15	perception.ab,ti.	170,405
16	rejection.ab,ti.	83,807
17	1 or 2 or 3 or 4 or 5 or 6 or 7 or 8 or 9 or 10 or 11 or 12 or 13 or 14 or 15 or 16	721,967
18	"Dermal leishman*".ab,ti.	561
19	"cutaneous leishman*".ab,ti.	7,526
20	oriental sore.ab,ti.	79
21	Uta.ab,ti.	557
22	Chiclero ulcer.ab,ti.	3
23	tropical sore.ab,ti.	0
24	Bagdad boil.ab,ti.	1
25	Baghdad boil.ab,ti.	2
26	Bauer ulcer.ab,ti.	0
27	Delhi boil.ab,ti.	1
28	Aleppo boil.ab,ti.	10
29	Aleppo button.ab,ti.	0
30	Jericho boil.ab,ti.	1
31	one year sore.ab,ti.	1
32	one year ulcer.ab,ti.	4
33	"tegumentary leishman*".ab,ti.	587
34	Biskra button.ab,ti.	1
35	Biskra nodule.ab,ti.	0
36	Calcutta ulcer.ab,ti.	0
37	Jericho button.ab,ti.	0

38	Kandahar sore.ab,ti.	0
39	Lahore sore.ab,ti.	0
40	Oriental button.ab,ti.	5
41	Pian bois.ab,ti.	4
42	Old World leishmaniasis.ab,ti.	38
43	"Mucosal Leishman*".ab,ti.	350
44	"Mucocutaneous Leishman*".ab,ti.	523
45	"muco cutaneous Leishman*".ab,ti.	31
46	espundia.ab,ti.	31
47	"nasopharyngeal Leishman*".ab,ti.	0
48	New World leishmaniasis.ab,ti.	49
49	American leishmaniasis.ab,ti.	86
50	leishmaniasis americana.ab,ti.	19
51	18 or 19 or 20 or 21 or 22 or 23 or 24 or 25 or 26 or 27 or 28 or 29 or 30 or 31 or 32 or 33 or 34 or 35 or 36 or 37 or 38 or 39 or 40 or 41 or 42 or 43 or 44 or 45 or 46 or 47 or 48 or 49 or 50	9,687
52	17 and 51	487
53	limit 52 to (english or portuguese or spanish)	451

"Stigma*".ab,ti.
"discriminat*".ab,ti.
"stereotyp*".ab,ti.
negative attitude.ab,ti.
psychosocial impact.ab,ti.
psychosocial burden.ab,ti.
social consequences.ab,ti.
stigmatizing effect.ab,ti.
"scar*".ab,ti.
"disfigure*".ab,ti.
self-stigma.ab,ti.
label avoidance.ab,ti.
disgrace.ab,ti.
shame.ab,ti.
perception.ab,ti.
rejection.ab,ti.
1 or 2 or 3 or 4 or 5 or 6 or 7 or 8 or 9 or 10 or 11 or 12 or 13 or 14 or 15 or 16
"Dermal leishman*".ab,ti.
"cutaneous leishman*".ab,ti.
oriental sore.ab,ti.
Uta.ab,ti.
Chiclero ulcer.ab,ti.
tropical sore.ab,ti.
Bagdad boil.ab,ti.
Baghdad boil.ab,ti.
Bauer ulcer.ab,ti.
Delhi boil.ab,ti.
Aleppo boil.ab,ti.
Aleppo button.ab,ti.
Jericho boil.ab,ti.
one year sore.ab,ti.
one year ulcer.ab,ti.
"tegumentary leishman*".ab,ti.
Biskra button.ab,ti.
Biskra nodule.ab,ti.
Calcutta ulcer.ab,ti.

Jericho button.ab,ti.
Kandahar sore.ab,ti.
Lahore sore.ab,ti.
Oriental button.ab,ti.
Pian bois.ab,ti.
Old World leishmaniasis.ab,ti.
"Mucosal Leishman*".ab,ti.
"Mucocutaneous Leishman*".ab,ti.
"muco cutaneous Leishman*".ab,ti.
espundia.ab,ti.
"nasopharyngeal Leishman*".ab,ti.
New World leishmaniasis.ab,ti.
American leishmaniasis.ab,ti.
leishmaniasis americana.ab,ti.
18 or 19 or 20 or 21 or 22 or 23 or 24 or 25 or 26 or 27 or 28 or 29 or 30 or 31 or 32 or 33 or 34 or 35 or 36 or 37 or 38 or 39 or 40 or 41 or 42 or 43 or 44 or 45 or 46 or 47 or 48 or 49 or 50
17 and 51
limit 52 to (english or portuguese or spanish)

https://ovidsp.ovid.com/athens/ovidweb.cgi?T=JS&NEWS=N&PAGE=main&SHAREDSEARCHID=4zN20dEhkm96FqvENxfG5qP29L31pDa7SIj4OdDMBSSEhrYDUtV7PDdIvGu21m2sU

Ovid MEDLINE(R) <1946 to February Week 5 2023>

1	"Stigma*".ab,ti.	40541
2	"discriminat*".ab,ti.	242711
3	"stereotyp*".ab,ti.	25345
4	negative attitude.ab,ti.	1709
5	psychosocial impact.ab,ti.	2247
6	psychosocial burden.ab,ti.	559
7	social consequences.ab,ti.	2855
8	stigmatizing effect.ab,ti.	27
9	"scar*".ab,ti.	171517
10	"disfigure*".ab,ti.	2661
11	self-stigma.ab,ti.	932
12	label avoidance.ab,ti.	15
13	disgrace.ab,ti.	152
14	shame.ab,ti.	5278
15	perception.ab,ti.	170405
16	rejection.ab,ti.	83807
17	1 or 2 or 3 or 4 or 5 or 6 or 7 or 8 or 9 or 10 or 11 or 12 or 13 or 14 or 15 or 16	721967
18	"Dermal leishman*".ab,ti.	561
19	"cutaneous leishman*".ab,ti.	7526
20	oriental sore.ab,ti.	79
21	Uta.ab,ti.	557
22	Chiclero ulcer.ab,ti.	3
23	tropical sore.ab,ti.	0
24	Bagdad boil.ab,ti.	1
25	Baghdad boil.ab,ti.	2
26	Bauer ulcer.ab,ti.	0

27 Delhi boil.ab,ti. 1
28 Aleppo boil.ab,ti. 10
29 Aleppo button.ab,ti. 0
30 Jericho boil.ab,ti. 1
31 one year sore.ab,ti. 1
32 one year ulcer.ab,ti. 4
33 "tegumentary leishman*".ab,ti. 587
34 Biskra button.ab,ti. 1
35 Biskra nodule.ab,ti. 0
36 Calcutta ulcer.ab,ti. 0
37 Jericho button.ab,ti. 0
38 Kandahar sore.ab,ti. 0
39 Lahore sore.ab,ti. 0
40 Oriental button.ab,ti. 5
41 Pian bois.ab,ti.4
42 Old World leishmaniasis.ab,ti. 38
43 "Mucosal Leishman*".ab,ti. 350
44 "Mucocutaneous Leishman*".ab,ti. 523
45 "muco cutaneous Leishman*".ab,ti. 31
46 espundia.ab,ti.31
47 "nasopharyngeal Leishman*".ab,ti. 0
48 New World leishmaniasis.ab,ti. 49
49 American leishmaniasis.ab,ti. 86
50 leishmaniasis americana.ab,ti. 19
51 18 or 19 or 20 or 21 or 22 or 23 or 24 or 25 or 26 or 27 or 28 or 29 or 30 or 31 or 32 or 33 or 34 or 35 or 36 or 37 or 38 or 39 or 40 or 41 or 42 or 43 or 44 or 45 or 46 or 47 or 48 or 49 or 50 9687
52 17 and 51 487
53 limit 52 to (english or portuguese or spanish) 451

PubMed search

Search number	Query	Filters	Search Details	Results	Time
3	((((((((((((((((Stigma*[Title/Abstract]) OR (discriminat*[Title/Abstract])) OR (stereotyp*[Title/Abstract])) OR (negative attitude[Title/Abstract])) OR (psychosocial impact[Title/Abstract])) OR (psychosocial burden[Title/Abstract])) OR (social consequences[Title/Abstract])) OR (stigmatizing effect[Title/Abstract])) OR (scar*[Title/Abstract])) OR (disfigure*[Title/Abstract])) OR (self-stigma[Title/Abstract])) OR (label avoidance[Title/Abstract])) OR (disgrace[Title/Abstract])) OR (shame[Title/Abstract])) OR (perception[Title/Abstract])) OR (rejection[Title/Abstract]) AND (english[Filter] OR portuguese[Filter] OR spanish[Filter])) AND (((((((((((((((((((((((((((((((((Dermal leishman*[Title/Abstract]) OR (cutaneous leishman*[Title/Abstract])) OR (oriental sore[Title/Abstract])) OR (Uta[Title/Abstract])) OR (Chiclero ulcer[Title/Abstract])) OR (tropical sore[Title/Abstract])) OR (Bagdad boil[Title/Abstract])) OR (Baghdad boil[Title/Abstract])) OR (Bauer ulcer[Title/Abstract])) OR (Delhi boil[Title/Abstract])) OR (Aleppo boil[Title/Abstract])) OR (Aleppo button[Title/Abstract])) OR (Jericho boil[Title/Abstract])) OR (one year sore[Title/Abstract])) OR (one year ulcer[Title/Abstract])) OR (tegumentary leishman*[Title/Abstract])) OR (Biskra button[Title/Abstract])) OR (Biskra nodule[Title/Abstract])) OR (Calcutta ulcer[Title/Abstract])) OR (Jericho button[Title/Abstract])) OR (Kandahar sore[Title/Abstract])) OR (Lahore sore[Title/Abstract])) OR (Oriental button[Title/Abstract])) OR (Pian bois[Title/Abstract])) OR (Old World leishmaniasis[Title/Abstract])) OR (Mucosal Leishman*[Title/Abstract])) OR (Mucocutaneous Leishman*[Title/Abstract])) OR (muco cutaneous Leishman*[Title/Abstract])) OR (espundia[Title/Abstract])) OR (nasopharyngeal Leishman*[Title/Abstract])) OR (New World leishmaniasis[Title/Abstract])) OR (American leishmaniasis[Title/Abstract])) OR (leishmaniasis americana[Title/Abstract]) AND (english[Filter] OR portuguese[Filter] OR spanish[Filter]))	English, Portuguese, Spanish	(("stigma*"[Title/Abstract] OR "discriminat*"[Title/Abstract] OR "stereotyp*"[Title/Abstract] OR "negative attitude"[Title/Abstract] OR "psychosocial impact"[Title/Abstract] OR "psychosocial burden"[Title/Abstract] OR "social consequences"[Title/Abstract] OR "stigmatizing effect"[Title/Abstract] OR "scar*"[Title/Abstract] OR "disfigure*"[Title/Abstract] OR "self-stigma"[Title/Abstract] OR "label avoidance"[Title/Abstract] OR "disgrace"[Title/Abstract] OR "shame"[Title/Abstract] OR "perception"[Title/Abstract] OR "rejection"[Title/Abstract]) AND ("english"[Language] OR "portuguese"[Language] OR "spanish"[Language]) AND (("dermal leishman*"[Title/Abstract] OR "cutaneous leishman*"[Title/Abstract] OR "oriental sore"[Title/Abstract] OR "Uta"[Title/Abstract] OR "chiclero ulcer"[Title/Abstract] OR (("tropic"[All Fields] OR "tropical"[All Fields] OR "tropicalization"[All Fields] OR "tropically"[All Fields] OR "tropics"[All Fields]) AND "sore"[Title/Abstract]) OR "bagdad boil"[Title/Abstract] OR "baghdad boil"[Title/Abstract] OR (("bauer"[All Fields] OR "bauer s"[All Fields]) AND "ulcer"[Title/Abstract]) OR "delhi boil"[Title/Abstract] OR "aleppo boil"[Title/Abstract] OR ("Aleppo"[All Fields] AND "button"[Title/Abstract]) OR ("Jericho"[All Fields] AND "boil"[Title/Abstract]) OR (("one"[All Fields] AND "year"[All Fields]) AND "sore"[Title/Abstract]) OR (("one"[All Fields] AND "year"[All Fields]) AND "ulcer"[Title/Abstract]) OR "tegumentary leishman*"[Title/Abstract] OR "biskra button"[Title/Abstract] OR ("Biskra"[All Fields] AND "nodule"[Title/Abstract]) OR ("calcutta"[All Fields] AND "ulcer"[Title/Abstract]) OR ("Jericho"[All Fields] AND "button"[Title/Abstract]) OR ("Kandahar"[All Fields] AND "sore"[Title/Abstract]) OR ("Lahore"[All Fields] AND "sore"[Title/Abstract]) OR "oriental button"[Title/Abstract] OR "pian bois"[Title/Abstract] OR "old world leishmaniasis"[Title/Abstract] OR "mucosal leishman*"[Title/Abstract] OR "mucocutaneous leishman*"[Title/Abstract] OR "muco cutaneous leishman*"[Title/Abstract] OR "espundia"[Title/Abstract] OR (("nasopharynx"[MeSH Terms] OR "nasopharynx"[All Fields] OR "nasopharyngeal"[All Fields]) AND "leishman*"[Title/Abstract]) OR "new world leishmaniasis"[Title/Abstract] OR "american leishmaniasis"[Title/Abstract] OR "leishmaniasis americana"[Title/Abstract]) AND ("english"[Language] OR "portuguese"[Language] OR "spanish"[Language]))) AND (english[Filter] OR portuguese[Filter] OR spanish[Filter])	687	23:01:23
2	((((((((((((((((((((((((((((((((Dermal leishman*[Title/Abstract]) OR (cutaneous leishman*[Title/Abstract])) OR (oriental sore[Title/Abstract])) OR (Uta[Title/Abstract])) OR (Chiclero ulcer[Title/Abstract])) OR (tropical sore[Title/Abstract])) OR (Bagdad boil[Title/Abstract])) OR (Baghdad boil[Title/Abstract])) OR (Bauer ulcer[Title/Abstract])) OR (Delhi boil[Title/Abstract])) OR (Aleppo boil[Title/Abstract])) OR (Aleppo button[Title/Abstract])) OR (Jericho boil[Title/Abstract])) OR (one year sore[Title/Abstract])) OR (one year ulcer[Title/Abstract])) OR (tegumentary leishman*[Title/Abstract])) OR (Biskra button[Title/Abstract])) OR (Biskra nodule[Title/Abstract])) OR (Calcutta ulcer[Title/Abstract])) OR (Jericho button[Title/Abstract])) OR (Kandahar sore[Title/Abstract])) OR (Lahore sore[Title/Abstract])) OR (Oriental button[Title/Abstract])) OR (Pian bois[Title/Abstract])) OR (Old World leishmaniasis[Title/Abstract])) OR (Mucosal Leishman*[Title/Abstract])) OR (Mucocutaneous Leishman*[Title/Abstract])) OR (muco cutaneous Leishman*[Title/Abstract])) OR (espundia[Title/Abstract])) OR (nasopharyngeal Leishman*[Title/Abstract])) OR (New World leishmaniasis[Title/Abstract])) OR (American leishmaniasis[Title/Abstract])) OR (leishmaniasis americana[Title/Abstract])	English, Portuguese, Spanish	("dermal leishman*"[Title/Abstract] OR "cutaneous leishman*"[Title/Abstract] OR "oriental sore"[Title/Abstract] OR "Uta"[Title/Abstract] OR "chiclero ulcer"[Title/Abstract] OR (("tropic"[All Fields] OR "tropical"[All Fields] OR "tropicalization"[All Fields] OR "tropically"[All Fields] OR "tropics"[All Fields]) AND "sore"[Title/Abstract]) OR "bagdad boil"[Title/Abstract] OR "baghdad boil"[Title/Abstract] OR (("bauer"[All Fields] OR "bauer s"[All Fields]) AND "ulcer"[Title/Abstract]) OR "delhi boil"[Title/Abstract] OR "aleppo boil"[Title/Abstract] OR ("Aleppo"[All Fields] AND "button"[Title/Abstract]) OR ("Jericho"[All Fields] AND "boil"[Title/Abstract]) OR (("one"[All Fields] AND "year"[All Fields]) AND "sore"[Title/Abstract]) OR (("one"[All Fields] AND "year"[All Fields]) AND "ulcer"[Title/Abstract]) OR "tegumentary leishman*"[Title/Abstract] OR "biskra button"[Title/Abstract] OR ("Biskra"[All Fields] AND "nodule"[Title/Abstract]) OR ("calcutta"[All Fields] AND "ulcer"[Title/Abstract]) OR ("Jericho"[All Fields] AND "button"[Title/Abstract]) OR ("Kandahar"[All Fields] AND "sore"[Title/Abstract]) OR ("Lahore"[All Fields] AND "sore"[Title/Abstract]) OR "oriental button"[Title/Abstract] OR "pian bois"[Title/Abstract] OR "old world leishmaniasis"[Title/Abstract] OR "mucosal leishman*"[Title/Abstract] OR "mucocutaneous leishman*"[Title/Abstract] OR "muco cutaneous leishman*"[Title/Abstract] OR "espundia"[Title/Abstract] OR (("nasopharynx"[MeSH Terms] OR "nasopharynx"[All Fields] OR "nasopharyngeal"[All Fields]) AND "leishman*"[Title/Abstract]) OR "new world leishmaniasis"[Title/Abstract] OR "american leishmaniasis"[Title/Abstract] OR "leishmaniasis americana"[Title/Abstract]) AND (english[Filter] OR portuguese[Filter] OR spanish[Filter])	13,525	23:01:05
1	(((((((((((((((Stigma*[Title/Abstract]) OR (discriminat*[Title/Abstract])) OR (stereotyp*[Title/Abstract])) OR (negative attitude[Title/Abstract])) OR (psychosocial impact[Title/Abstract])) OR (psychosocial burden[Title/Abstract])) OR (social consequences[Title/Abstract])) OR (stigmatizing effect[Title/Abstract])) OR (scar*[Title/Abstract])) OR (disfigure*[Title/Abstract])) OR (self-stigma[Title/Abstract])) OR (label avoidance[Title/Abstract])) OR (disgrace[Title/Abstract])) OR (shame[Title/Abstract])) OR (perception[Title/Abstract])) OR (rejection[Title/Abstract])	English, Portuguese, Spanish	("stigma*"[Title/Abstract] OR "discriminat*"[Title/Abstract] OR "stereotyp*"[Title/Abstract] OR "negative attitude"[Title/Abstract] OR "psychosocial impact"[Title/Abstract] OR "psychosocial burden"[Title/Abstract] OR "social consequences"[Title/Abstract] OR "stigmatizing effect"[Title/Abstract] OR "scar*"[Title/Abstract] OR "disfigure*"[Title/Abstract] OR "self-stigma"[Title/Abstract] OR "label avoidance"[Title/Abstract] OR "disgrace"[Title/Abstract] OR "shame"[Title/Abstract] OR "perception"[Title/Abstract] OR "rejection"[Title/Abstract]) AND (english[Filter] OR portuguese[Filter] OR spanish[Filter])	830,380	22:57:16

Scopus search
10/03/2023

TITLE-ABS-KEY (("Stigma*") OR ("discriminat*") OR ("stereotyp*") OR ("negative attitude") OR ("psychosocial impact") OR ("psychosocial burden") OR ("social consequences") OR ("stigmatizing effect") OR ("scar*") OR ("disfigure*") OR ("self-stigma") OR ("label avoidance") OR ("disgrace") OR ("shame") OR ("perception") OR ("rejection") AND ("Dermal leishman*") OR ("cutaneous leishman*") OR ("oriental sore") OR ("Uta") OR ("Chiclero ulcer") OR ("tropical sore") OR ("Bagdad boil") OR ("Baghdad boil") OR ("Bauer ulcer") OR ("Delhi boil") OR ("Aleppo boil") OR ("Aleppo button") OR ("Jericho boil") OR ("one year sore") OR ("one year ulcer") OR ("tegumentary leishman*") OR ("Biskra button") OR ("Biskra nodule") OR ("Calcutta ulcer") OR ("Jericho button") OR ("Kandahar sore") OR ("Lahore sore") OR ("Oriental button") OR ("Pian bois") OR ("Old World leishmaniasis") OR ("Mucosal Leishman*") OR ("Mucocutaneous Leishman*") OR ("muco cutaneous Leishman*") OR ("espundia") OR ("nasopharyngeal Leishman*") OR ("New World leishmaniasis") OR ("American leishmaniasis") OR ("leishmaniasis americana")) AND (LIMIT-TO (LANGUAGE , "English") OR LIMIT-TO (LANGUAGE , "Spanish") OR LIMIT-TO (LANGUAGE , "Portuguese"))

Results 722 documents

Trip database search
10/03/2023

("dermal Leishma*" OR "cutaneous leishma*" OR "oriental sore" OR uta OR "chiclero ulcer" OR "tropical sore" OR "Baghdad boil" OR "Bauer ulcer" OR "delhi boil" OR "Aleppo boil" OR "Aleppo button" OR "Jericho boil" OR "balkh sore" OR "one year sore" OR "one year ulcer" OR "tegumentary leishman*" OR "Mucosal Leishman*" OR "Mucocutaneous Leishman*" OR "Muco cutaneous Leishman*" OR espundia OR "Nasopharyngeal leishman*" OR "new world leishman*" OR "american leishmaniasis" OR "leishmaniasis americana" OR "Biskra button" OR "Biskra nodule" OR "Calcutta ulcer" OR "Jericho button" OR "Kandahar sore" OR "Lahore sore" OR "Oriental button" OR "Pian bois" OR "Old World leishmaniasis") AND (stigma* OR discriminat* OR stereotyp* OR "negative attitude" OR psychological OR psychosocial OR "social consequences" OR scar* OR disfigur* OR self-stigma OR "label avoidance" OR disgrace OR shame OR perception OR rejection)

Results - 79

Web of Science Search Strategy (v0.1)

Database: All Databases

Entitlements:

- WOS: 1970 to 2023
- BCI: 1969 to 2023
- CCC: 1998 to 2023
- DRCI: 2008 to 2023
- DIIDW: 2008 to 2023
- KJD: 1980 to 2023
- MEDLINE: 1950 to 2023
- PPRN: 1991 to 2023
- SCIELO: 2002 to 2023
- ZOOREC: 2008 to 2023

Searches:

1: (TI=(Stigma*OR discriminat* OR stereotyp* OR negative attitude OR psychosocial impact OR psychosocial burden OR social consequences OR stigmatizing effect OR scar* OR disfigure* OR self-stigma OR label avoidance OR disgraice OR shape OR perception OR rejection)) OR AB=(Stigma*OR discriminat* OR stereotyp* OR negative attitude OR psychosocial impact OR psychosocial burden OR social consequences OR stigmatizing effect OR scar* OR disfigure* OR self-stigma OR label avoidance OR disgraice OR shape OR perception OR rejection) Date Run: Fri Mar 10 2023 13:23:18 GMT+0530 (+0530) Results: 8497247

2: (TI=(Dermal leishman* OR cutaneous leishman* OR oriental sore OR Uta OR chiclera ulcer OR tropical sore OR bagged boil OR bighead boil OR Bauer ulcer OR Delhi boil OR Aleppo boil OR Aleppo button OR jebicha boil OR one year sore OR one year ulcer tegumentary leishman* OR bisra button OR bisra nodule OR calcuate ulcer OR jebicha button OR Kandahar sore OR labors sore OR Oriental button OR plan boss OR Old World leishmaniasis OR Mucosal Leishman* OR Mucocutaneous Leishman* OR muco cutaneous Leishman* OR eklundia OR nasopharyngeal Leishman* OR New World leishmaniasis OR American leishmaniasis OR leishmaniasis americana)) OR AB=(Dermal leishman* OR cutaneous leishman* OR oriental sore OR Uta OR chiclera ulcer OR tropical sore OR bagged boil OR bighead boil OR Bauer ulcer OR Delhi boil OR Aleppo boil OR Aleppo button OR jebicha boil OR one year sore OR one year ulcer tegumentary leishman* OR bisra button OR bisra nodule OR calcuate ulcer OR jebicha button OR Kandahar sore OR labors sore OR Oriental button OR plan boss OR Old

World leishmaniasis OR Mucosal Leishman* OR Mucocutaneous Leishman* OR muco cutaneous Leishman* OR eklundia OR nasopharyngeal Leishman* OR New World leishmaniasis OR American leishmaniasis OR leishmaniasis americana) Date Run: Fri Mar 10 2023 13:28:23 GMT+0530 (+0530) Results: 36382

3: #1 AND #2 Date Run: Fri Mar 10 2023 13:30:12 GMT+0530 (+0530) Results: 2577

4: #1 AND #2 and Preprint Citation Index or KCI-Korean Journal Database or SciELO Citation Index or Current Contents Connect or Zoological Record or Data Citation Index or BIOSIS Citation Index or Web of Science Core Collection or Derwent Innovations Index (Database) Date Run: Fri Mar 10 2023 13:33:53 GMT+0530 (+0530) Results: 2433

5: #1 AND #2 and Preprint Citation Index or KCI-Korean Journal Database or SciELO Citation Index or Current Contents Connect or Zoological Record or Data Citation Index or BIOSIS Citation Index or Web of Science Core Collection or Derwent Innovations Index (Database) and English or Spanish or Portuguese (Languages) Date Run: Fri Mar 10 2023 13:34:16 GMT+0530 (+0530) Results: 2386
