# Supplementary material for: Stigma associated with cutaneous and mucocutaneous leishmaniasis: A systematic review
Source: PLoS Negl Trop Dis. 2023 Dec 28;17(12):e0011818. doi: 10.1371/journal.pntd.0011818 (PMC10781107; doi:10.1371/journal.pntd.0011818)
Supplement: Attachment — Submitted filename: Response to reviewers.docx [file pntd.0011818.s003.docx]

**Response to the reviewers**

We thank all the reviewers for their constructive comments. The responses to all comments are listed below.

| Reviewer #1 | |
| --- | --- |
| Methods | |
| The methods are clear and articulate; appropriate design, sufficient sample size and correct statistical analyses were employed. The study was registered. | We thank the reviewer for this comment |
| Results | |
| The analysis plan matches the actual analysis; results clearly presented; however the figures attached in the appendices seem blurry, It would be good to use clearer figures as in the body of the manuscript | We thank the reviewer for this comment. We have now provided images according to the journal guidelines |
| Conclusions |  |
| The conclusion is subtle and clearly describes a summary of the data, limitations, public health implications of the study. | We thank the reviewer for this comment |
| Summary and General Comments | |
| I am concerned that this same study has already been published as below <https://doi.org/10.1371/journal.pone.0285663> <http://www.ncbi.nlm.nih.gov/pmc/articles/pmc10174477/>  Could there be any reason why this paper is undergoing a review for publication again? or could this be an issue of ethical concern | The referenced article is the protocol of this systematic review, which was published prior to the review. We published our protocol to uphold the expected standards of transparency and rigour for a systematic review. Here we are publishing the final review using the protocol exactly as described in the first paper. |

| Reviewer #2: | |
| --- | --- |
| Methods | |
| The objectives of the study are clearly articulated and the study design is appropriate to address the stated objectives | We thank the reviewer for this comment |
| Results | |
| The results are clearly and completely presented. Figures and tables included are sufficient | We thank the reviewer for this comment |
| Conclusions |  |
| The conclusions are supported by the data presented | We thank the reviewer for this comment |
| Summary and General Comments | |
| The bibliographic search article includes an appropriate methodology and all articles included in the manuscript are referenced. Nevertheless, a discrepancy is found between the included articles for the synthesis referred to in the text (16 articles, lines 157, 158, 177, …) and those of studies included in the review in Figure 1 (20 articles). | We thank the reviewer for highlighting this error. We have now revised Figure 1 to reflect the correct number. |
| Specific comments | |
| Do not include abbreviations in the summary, or do so for all concepts introduced (MMAT, ...). | This is now corrected. Please see line 33 |
| Introduction | |
| include a more recent WHO reference regarding the clinical manifestations of leishmaniasis and its geographical distribution. In any case, as it is in general, it should not be limited, like the one included (ref. 1), to a cutaneous leishmaniasis reference nor to the eastern Mediterranean region. Also, usually three main clinical manifestations of leishmaniasis are considered: cutaneous, mucocutaneous and visceral. PKDL is a known complication of VL, as LCD or other complications are from LC. Perhaps because of this, the authors do not include studies that specifically deal with PKDL. | The reference had been updated and the introduction has been modified to reflect the 3 clinical manifestations. Please see line 73-77 |
| Lines 99 and 103: In line 99 it is mentioned the study of Bos et al. (ref. 15), in relation to the stigma types related to the impact of public stigma, but at the end of the phrase (line 103) there are included three references (15,16,17). Should it be replaced “Bos et al.” by “Some authors”? | Thank you for raising this. We have amended the sentence to make it clear and precise. We have included the two references that originally present this self-stigma classification [17, 18] and have also cited Bos et al [16] who have included the same classification referring to the original source, as we state that we use Bos el for conceptualization of stigma in this study (Line 104-105) |
| Methods – Eligibility criteria | |
| Change “Qualitative, quantitative, cross-sectional, mixed-method, and ethnographic/anthropological studies were included, but articles targeting veterinary studies, vector studies, laboratory-based research, clinical trials, diagnostic or treatment methods for CL and MCL and articles that explore stigma only in VL or PKDL were excluded.” by “Qualitative, quantitative, cross-sectional, mixed-method, and ethnographic/anthropological studies on human leishmaniasis were included, but articles targeting laboratory-based research, clinical trials, diagnostic or treatment methods for CL and MCL and articles that explore stigma only in VL or PKDL were excluded.” | This sentence was revised as suggested (Line 137-140) |
| Include some explanation on the types of studies: qualitative, quantitative, cross-sectional, mixed-method, and ethnographic/anthropologica. | The sentence was revised to enhance the clarity in describing the types of studies. (Line 137-140) |
| Study design |  |
| Line 178: is reference 37 included among the mixed methods studies? | This was revised and removed |
| Line 179: Replace “The majority” by “Five out of 11” | This is corrected (Line 188-189) |
| Results |  |
| Table 1 and 2: include the reference number next to the author, as done in the table 3. | This is corrected (Table 1 & 2) |
| Figure 3: The font size of the figure could be enlarged? | The figure was formatted to fit to journal requirements |
| Starting from line 245 of the results, and to improve tracking, include in the text references next to the authors and remove the references from the end of the sentence. Follow the examples, line 245: Studies by Bennis, Thys, et al. in Morocco [45,46] … for divorce.” and line 248 “Studies by Stewart & Brieger [37] and Reithinger et al. [36] in Afghanistan …. because of CL”. | This is corrected in relevant places |
| Line 408: Replace “Multiple” by “Two”. | This is corrected. Please see Line 415 |
| Discussion |  |
| Given that the authors previously published an article on a protocol for a systematic review of the stigma associated with cutaneous leishmaniasis and mucocutaneous leishmaniasis, it would be of interest to include some discussion of the gaps and difficulties encountered in the application of said protocol in the bibliographic search just carried out and how they could be solved. | We conducted the search after the protocol paper was accepted (in March 2023) even though the paper was published in May 2023. The bibliographic search was conducted as it is reported in the protocol. The search strings in the protocol were refined extensively before including them in the protocol paper. Hence, we did not face any particular challenge nor were any gaps to report specifically in the discussion. |
| Include complete reference for reference 16. | This is corrected |
| Review the reference 22. | This is corrected |
| Reference 38 cannot be consulted with the information included. | This is now corrected |
