# Supplementary material for: Stigma associated with cutaneous and mucocutaneous leishmaniasis: A systematic review
Source: PLoS Negl Trop Dis. 2023 Dec 28;17(12):e0011818. doi: 10.1371/journal.pntd.0011818 (PMC10781107; doi:10.1371/journal.pntd.0011818)
Supplement: Attachment — Submitted filename: Response to Editor.docx [file pntd.0011818.s005.docx]

| Comment | Response |
| --- | --- |
| (Line 1) Remove “The” | This is corrected |
| Abstract - Structure as background, Methods, Results, and conclusions | This is corrected |
| Introduction (Line 78) - Cite | The reference was added |
| (Line 120) – “aims” rewrite in past tense | This is corrected |
| (Line 134) - add “In this Journal [PLoS ONE]” | This is corrected |
| (Line 136-145) Eligibility criteria - Separate sub-headings for inclusion and exclusion criteria | This is corrected |
| (Line 138 – 141) - A lot of duplication here | This is corrected |
| (Line 152) ‘…. And analysis” Separate section | This is corrected |
| Discussion – (Line 441-444) Quantity and categorise the stigma | The error was corrected, and as it is not possible to quantify stigma, the sentence revised (Line 438-439) |
| (Line 511-513) What are the strengths of the study? How were limitations addressed? | The suggestions were incorporated (Line 513-519) |

Response to editor

We thank the editor for their constructive comments. The responses to all comments are listed below.
